# A Model of Blood Pressure, Heart Rate, and Vaso-Vagal Responses Produced by Vestibulo-Sympathetic Activation

**DOI:** 10.3389/fnins.2016.00096

**Published:** 2016-03-31

**Authors:** Theodore Raphan, Bernard Cohen, Yongqing Xiang, Sergei B. Yakushin

**Affiliations:** ^1^Department of Computer and Information Science, Institute for Neural and Intelligent Systems, Brooklyn College, City University of New YorkNew York, NY, USA; ^2^Department of Neurology, Icahn School of Medicine at Mount SinaiNew York, NY, USA

**Keywords:** rat, vasovagal syncope, relaxation oscillator, baroreflex, pulse pressure, modeling and simulations

## Abstract

Blood Pressure (BP), comprised of recurrent systoles and diastoles, is controlled by central mechanisms to maintain blood flow. Periodic behavior of BP was modeled to study how peak amplitudes and frequencies of the systoles are modulated by vestibular activation. The model was implemented as a relaxation oscillator, driven by a central signal related to Desired BP. Relaxation oscillations were maintained by a second order system comprising two integrators and a threshold element in the feedback loop. The output signal related to BP was generated as a nonlinear function of the derivative of the first state variable, which is a summation of an input related to Desired BP, feedback from the states, and an input from the vestibular system into one of the feedback loops. This nonlinear function was structured to best simulate the shapes of systoles and diastoles, the relationship between BP and Heart Rate (HR) as well as the amplitude modulations of BP and Pulse Pressure. Increases in threshold in one of the feedback loops produced lower frequencies of HR, but generated large pulse pressures to maintain orthostasis, without generating a VasoVagal Response (VVR). Pulse pressures were considerably smaller in the anesthetized rats than during the simulations, but simulated pulse pressures were lowered by including saturation in the feedback loop. Stochastic changes in threshold maintained the compensatory Baroreflex Sensitivity. Sudden decreases in Desired BP elicited non-compensatory VVRs with smaller pulse pressures, consistent with experimental data. The model suggests that the Vestibular Sympathetic Reflex (VSR) modulates BP and HR of an oscillating system by manipulating parameters of the baroreflex feedback and the signals that maintain the oscillations. It also shows that a VVR is generated when the vestibular input triggers a marked reduction in Desired BP.

## Introduction

Fainting due to vasovagal syncope is a neurogenically induced transient loss of consciousness and body tone that can result in falls, fractures, and death (Gendelman et al., 1983; Moya et al., 2009). At present, treatment of frequent syncope using implanted pacemakers or beta blockers is often ineffective (Calkins, 1999; Sheldon, 1999; Sheldon et al., 2003a,b; Kapoor, 2008), because the underlying changes in the cardiovascular system that lead to the syncope are still incompletely understood (Sutton and Bloomfeld, 1999; Grubb, 2005). Therefore, identifying which factors trigger the Vaso-Vagal Response (VVR) that underlies Vaso-Vagal Syncope, could be important in predicting their onset and managing the condition.

Multiresolution analysis with wavelets demonstrated that there was increased power in low frequency modulations of blood pressure (BP) before an episode of Vaso-Vagal Syncope in a human fainter (Nowak et al., 2009). There have also been suggestions that BP oscillations during tilt testing are a predictive marker for Vaso-Vagal syncope (Hausenloy et al., 2009). Recently, VVRs have been generated in anesthetized rats by repetitively activating the Vestibulo-Sympathetic Reflex (VSR), using sinusoidal galvanic vestibular stimulation (sGVS), 70° head-up tilts and ±70° oscillation in pitch (Cohen et al., 2011, 2013; Yakushin et al., 2014). Although anesthetized rats cannot faint, the changes in the rat cardiovascular system that reduce BP and Heart Rate (HR) during the VVR are the same as those that produce the neurogenically mediated, Vaso-Vagal Syncope in humans (Nowak et al., 2009).

The VVRs in rats were associated with increased power in the low frequency band (0.025–0.05 Hz) with synchronous oscillations in BP and HR, which we have termed VasoVagal Oscillations; higher frequencies rarely induced a VVR (Yakushin et al., 2014). A critical feature of VVRs in humans and rats is the simultaneous occurrence of both bradycardia and hypotension at the onset of the VVR (Lewis, 1932), which suggests a loss of baroreflex function (Thomson et al., 1997; Julu et al., 2003; Cohen et al., 2013). Consistent with this, there is also an immediate loss of the baroreflex-generated Muscle Sympathetic Nerve Activity (MSNA) at the onset of syncope (Morillo and Villar, 1997; Morillo et al., 1997; Mosqueda-Garcia et al., 1997). The pathophysiological mechanism and significance of the baroreflex disengagement in producing bradycardia and hypotension have been conjectured to be the basis for the VVR and for Vaso-Vagal Syncope, but no specific mechanism has been identified that could produce these changes. Before such a mechanism could be accepted, it would be necessary to produce a model that would have the following properties: (1) The systolic/diastolic oscillation should have an approximate triangular shape. (2) Parameters would have to be identified to show how the oscillations could be modified and (3) A theory for how the amplitudes and frequencies of these systolic/diastolic oscillations could be controlled by vestibular input. More importantly, for this study, an overall framework would have to be established to consider what induces a VVR.

The underlying purpose of this study was to develop a bottom up mathematical model that generated systoles and diastoles, which simulated those measured in the anesthetized rat. This enabled a modeling framework for studying the modulations in BP and HR and to define the parameters that might be important for generating VVRs. Other models of BP modulation have been developed to address these questions, but they generally have used input-output linear models (Julien, 2006). Such models do not apply to oscillating systems, however, and cannot explicate their control, which is inherently nonlinear (Ottesen, 2000; Olufsen et al., 2006; Ocon et al., 2011). Consequently, a model was developed using the conceptual framework of a relaxation oscillator that produces a non-sinusoidal repetitive output (Van der Pol and Van der Mark, 1927, 1928; Noble and Noble, 2011).

The second order system that realized the relaxation oscillator had states and nonlinear thresholding and saturation, consistent with feedback mechanisms characteristic of the baroreflex feedback. The model oscillations displayed systolic and diastolic behavior, which were sustained by an input related to Desired BP that sustained the systolic and diastolic transitions. The frequency and amplitude of the oscillations were modulated by introducing low frequency vestibular input into the feedback loop to test the hypothesis that vestibular control of the systolic-diastolic oscillations were accomplished through the baroreflex feedback. Simulations were performed to identify how specific parameters of the model affected the frequency and amplitude of the oscillations in BP and HR, as well as how modification of the parameters could simulate the behavior of the VVR. The model was further tested by comparing its predictions to experimental data on pulse pressure during normal and VVR. From this model-based analysis, hypotheses were formulated about the parameters of the system that caused a cessation of oscillations leading to VVRs and the structures in the central nervous system that might be involved in generating a VVR.

## Materials and methods

### Source of experimental data for model-data comparisons

The raw data on BP and HR variations that were utilized for comparison with the model predictions were reported in three publications where the Methods and number of animals studied were completely described (Cohen et al., 2011, 2013; Yakushin et al., 2014). However, it should be pointed out that new methods of analysis were developed for this paper so that model predictions and data could be compared. In particular, the relationship between variations in pulse pressure and systolic BP as well as the baroreflex sensitivity, which were compared to model predictions were critical tests of the model's predictive capabilities.

The surgical and experimental procedure, aspects of simulation, and collection and analysis of data were also described in detail in the above mentioned publications (Cohen et al., 2011, 2013; Yakushin et al., 2014). All surgical procedures and experiments were approved by the Institutional Care and Use Committee of the Icahn School of Medicine at Mount Sinai. In brief, the BP and HR data from isoflurane-anesthetized rats were used as a basis for comparison with the model predictions. These data were obtained from young adult, male Long-Evans rats (Harlan Laboratories, MA) anesthetized with isoflurane (4% induction, 2% maintenance with oxygen). Oscillations in BP and HR were generated by binaural, 3mA, 0.025 or 0.05 Hz sGVS that caused Vaso-Vagal Oscillations and VVRs. The rats were also tilted nose-up 60°–70° (0.91 g) and oscillated ±70° in pitch at 0.025 and 0.05 Hz. The BP was recorded from intra-aortic sensors (DSI, St. Paul, MN) and stored at a sampling rate of 1 KHz with 12-bit resolution using our data collection program. HR was derived from BP by detecting the systoles with a peak detection algorithm that we developed, and computing the inverse of the time difference between them. The model and the associated simulations were implemented using Matlab (Mathworks, Inc.).

### Methodological approach

The methodological approach was to model the BP variations by starting from a “microscopic” point of view at the level of generating a systolic-diastolic pulse. We then show how such a pulse can be repetitively maintained by a signal, which we have termed Desired Blood Pressure (Desired BP). By modeling the systolic-diastolic pulse as being generated by a relaxation oscillator, the triangular wave-shape of the pulse was similar to the recorded BP pulse and the repetitive oscillations were sustained. Similar approaches have been used to model the repetitive motion of the eyes during vestibular and optokinetic nystagmus as well as the repetitive motion of the foot during locomotion (Osaki et al., 2007, 2008; Xiang et al., 2007; Cho et al., 2010). We next determined whether changing key parameters that drive the model and are known to exist in the baroreflex feedback loop could predict the macroscopic behavior of the systolic BP variations as a function of vestibular input. We next tested the model against data to determine how the model might generate a VVR, which is a macroscopic behavior and that was induced in the experiments by vestibular activation (sGVS and tilts). We then tested the model to determine whether other emergent properties such as pulse pressure variations and baroreflex sensitivity were consistent with recorded data.

## Results

### Conceptual basis of the model

**Figure 1:**
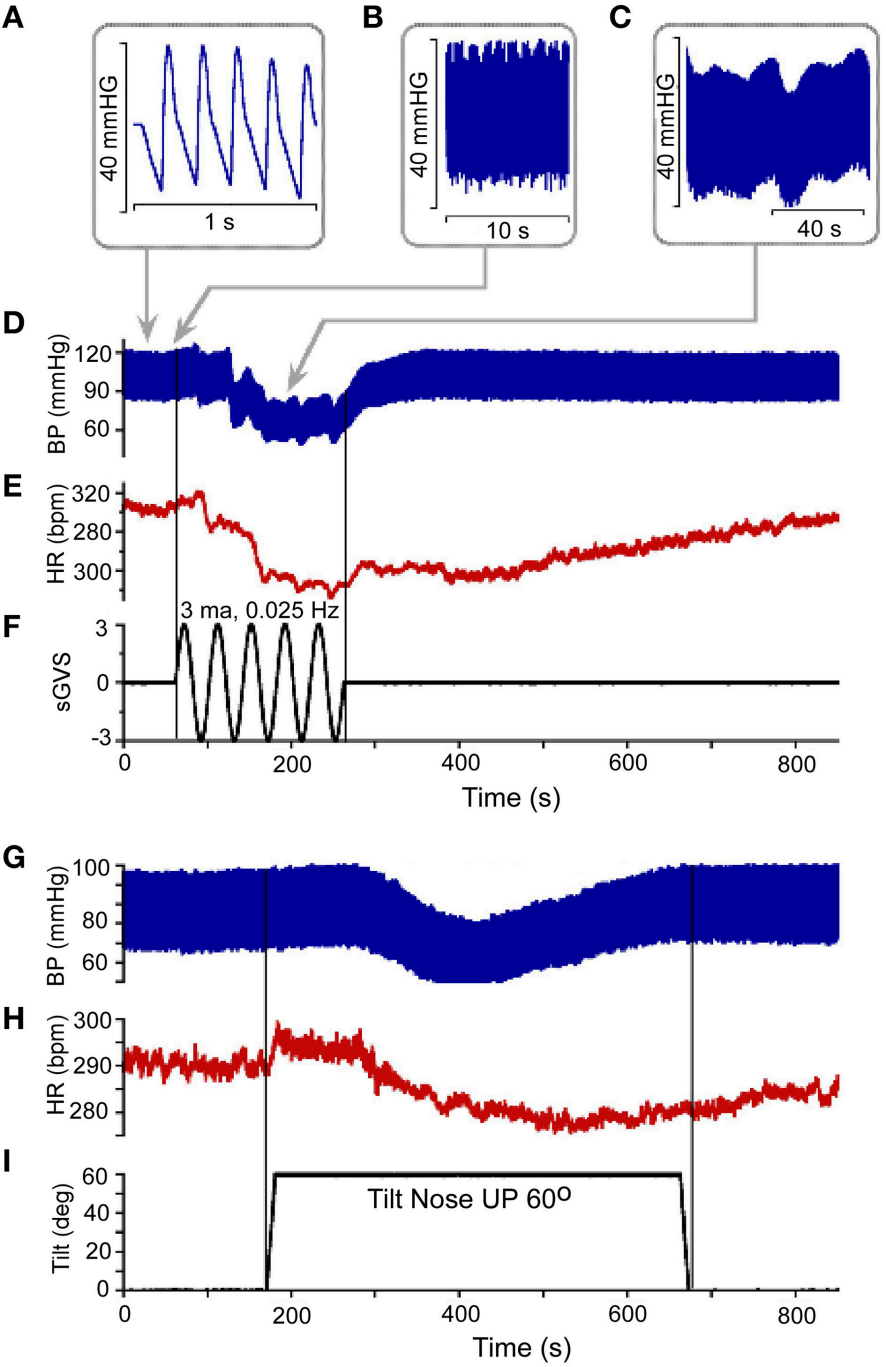
**(A)** Resting Blood Pressure response (BP) over 1 s time scale showing the triangular shapes of diastolic to systolic transitions. **(B,C)** BP variations over 10 s **(B)** and 40 s **(C)** scale. Vasovagal Response in BP **(D)** and HR **(E)** in response to ± 3 mA 0.025 Hz sinusoidal Galvanic Vestibular Stimulation (sGVS). This stimulus **(F)** generated a VVR, which is characterized by a transient decline in BP **(D)** followed by a decline in HR **(E)**. The two vertical lines represent the start and stop of stimulation, respectively. The low level of HR outlasted the low level of BP **(D,E)**. There was also a transient drop in BP **(G)** and HR **(H)** in response to nose up tilt of 60° **(I)**. The tilt up and back are shown by the two vertical lines, respectively. The transient drops in BP are generally slower than during sGVS, but the recovery follows a similar time course where HR rises to baseline values slower than BP **(G,H)**.

The model was derived from data showing that BP is a temporally repetitive waveform that can be analyzed at different time scales. At the smallest time scale (1 s) there are systolic to diastolic transitions whose waveform is approximately triangular in shape and can be reproduced by the relaxation oscillator developed in this study (Figure 1A). The idea that the heart oscillation can be described by a relaxation oscillator was first introduced by Van der Pol and Van der Mark (1928) and models based on this idea are discussed by Noble and Noble (2011). An important point of the model presented here is that the shape of the systolic/diastolic waveform is not determined strictly by the heart. It is determined by an internal model (relaxation oscillator) through a feedback neural network, which mimics the oscillation features of the heart. The feedback mechanisms implement closed loop control and then activate actuators through nonlinear mechanisms that control the constriction of the vascular beds, which has been referred to as peripheral vascular resistance. The feedback from the baroreceptors is compared with the output of the internal model to implement model-reference control. At a larger time scale (10 s), oscillations in systolic amplitude related to breathing can be observed (Figure 1B). There are also small amplitude very low frequency oscillations in the systolic and diastolic BP that have been termed Mayer waves (Mayer, 1876; Myers et al., 2001; Julien, 2006) as well as low frequency oscillations, which are associated with VVR's, which we have termed VasVagal Oscillations (Yakushin et al., 2014).

Vestibular system activation using sGVS at low frequencies (0.025 Hz) induces oscillations with strong second harmonics, the vasovagal oscillations, in addition to transient drops in BP, which are best analyzed over a much longer time scale (800 s; Figures 1C,D; Yakushin et al., 2014). The transient decline in systolic and diastolic BP in response to sGVS, the VVR, is typically initiated in susceptible animals following a period of low frequency vasovagal oscillations (Figure 1D). The VVR is accompanied by an almost synchronous decline in HR (Figure 1E). The VVR also can be initiated immediately at the start of stimulation or can be delayed up to minutes of stimulation accompanied by increased amplitudes of vasovagal oscillations (Yakushin et al., 2014). The transient drop in BP is followed by a slow rise back to steady state levels (Figure 1D). The transient return time course is variable, returning to steady state when stimulation is stopped (Figure 1F), but often the return to steady state occurs while the stimulation is ongoing (Yakushin et al., 2014). HR returns to steady state levels over a longer time course than does BP (cf: Figures 1D,E). Similar VVRs were generated by 70° head-up tilt from a prone position (Figures 1G–I), although there are differences between effects of tilt and sGVS. BP and HR begin to oscillate following the tilt and then drop, generating a VVR, but the transient drops in BP and HR are slower than during sGVS (Figures 1G,H).

**Figure 2:**
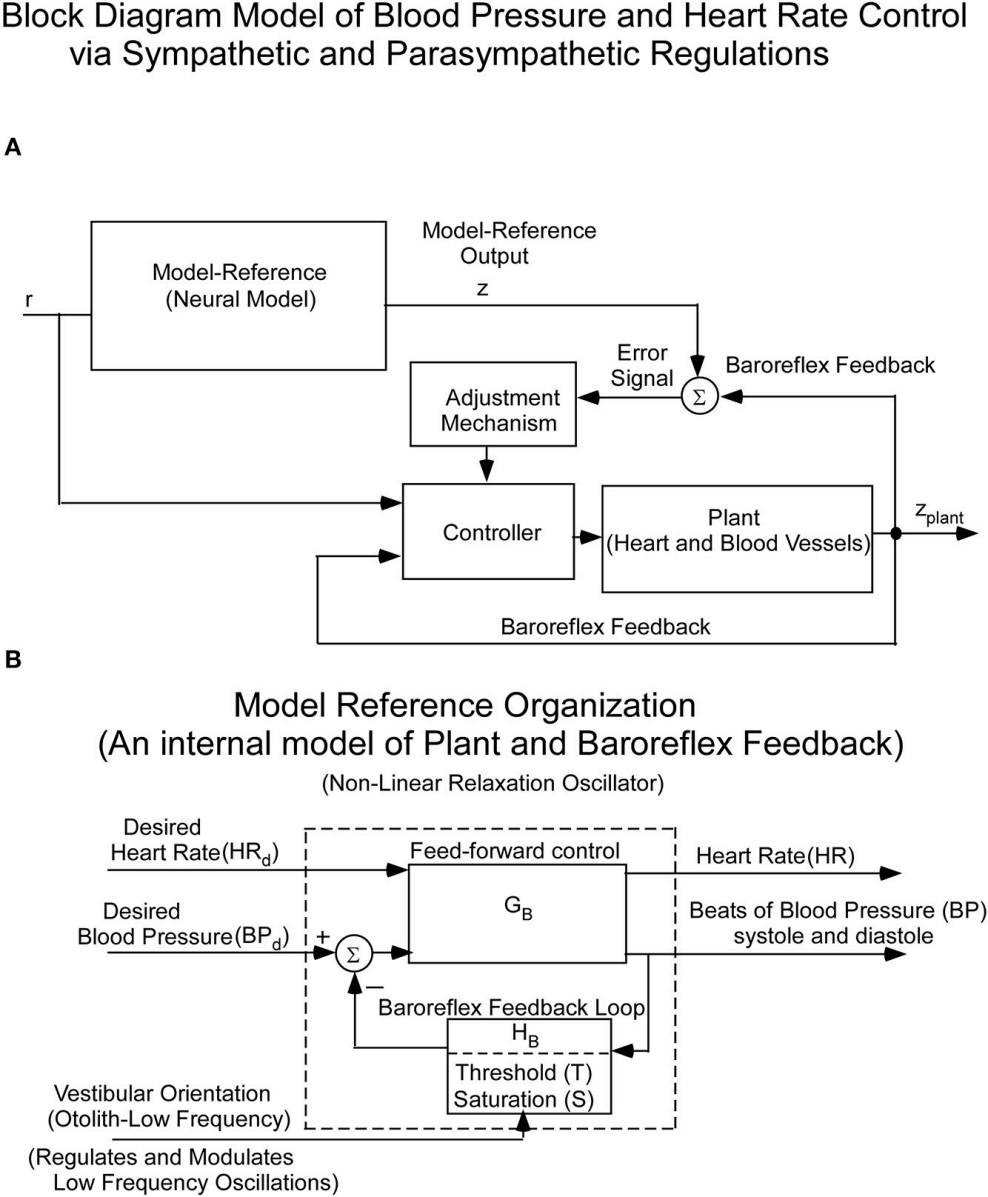
**(A)** Block diagram of the hypothesized Model Reference Adaptive Control (MRAC) of BP and HR. The input (**r**) is a constant input to the Neural Reference Model, which is an internal model of the dynamics of the heart blood vessels (plant), and baroreflex feedback. The output of this internal model reference is **z**. The model output (**z**) is compared with the plant (Heart and Blood Vessel) output via feedback sensors (Baroreflex). The error drives an adjustment mechanism for the controller that drives the heart and blood vessels (Plant). **(B)** Organization of the Neural Model Reference, which is an internal model of an oscillator that controls the beating heart and blood pressure oscillations. The oscillations of the internal model are controlled by Desired Blood Pressure, Desired Heart Rate, and vestibular (otolith) input.

Since the cardiovascular system, which is an inherently oscillating system, must adapt quickly to changes in vestibular input, it cannot be controlled in a linear closed loop input-output manner. Rather, we and others hypothesized that it is driven by Model Reference Adaptive Control (**MRAC**; Horrocks, 1964; White, 1966; Landau, 1979) using an internal model of BP control (Figure 2A). That is, there is an internal neural model whose output is compared to a feedback signal from baro-sensors that code BP (Figure 2A). This comparison generates an error signal whose control parameters are updated based on this error signal. The control parameters then converge to ideal values that cause the actual BP and HR to approximately match the response of the reference model. This classic model-reference design and conceptual basis has been used in this study to understand the sensory-based neural control of the cardiovascular system. The reference model whose predictions have been tested against data is described below. The block diagram organization of the model reference as a feedback control system, putting in evidence the Baroreflex Feedback Loop, with its threshold and saturation and vestibular otolithic input, is shown in Figure 2B.

### Model description

The slow and rapid BP changes that have approximately triangular waveforms are characteristic of a relaxation oscillator (van Der Pol, 1926; Van der Pol and Van der Mark, 1928; Raphan, 1976). The relaxation oscillations were postulated to be sustained by constant reference input signals, which were designated as Desired Blood Pressure (Desired BP) and Desired Heart Rate (Desired HR) through a feed-forward path, **G_B_** (Figure 2A). Desired BP and Desired HR were presumably determined by an overall system demand, but where such signals might originate is not known. For simplicity, we considered an oscillator driven by only Desired BP.

The model output, BP, is fed back through a system, **H_B_**, which mimics the function of the baroreflex, but probably involves other autonomic feedback loops. The output of **H_B_** is compared to Desired BP and the difference generates the error signal that stabilizes the oscillations as the system transits from the systolic to the diastolic phase. The feedback system, **H_B_**, is posited to contain a threshold, **T**, which governs the transitions from the systolic to diastolic phases and is set to sustain the oscillations at a particular frequency for a given Desired BP. The otolith system in the vestibular nuclei is posited to couple to this feedback control mechanism, regulating the feedback and the threshold, **T**.

The feedback system, **H_B_**, is postulated to mimic the baroreflex feedback loop, which begins at the baroreceptor sensors located in the carotid sinus and aortic arch (See Blessing, 1997) for summary). The afferent information is transmitted to Nucleus Tractus Solitarius (NTS) bilaterally, which excites the Caudal Ventro-Lateral Medulla (CVLM). In turn, CVLM inhibits the glutamatergic neurons in the Rostral Ventro-Lateral Medulla (RVLM; Neff et al., 1998; Sved et al., 2000, 2001; Schreihofer and Guyenet, 2002; Sugiyama et al., 2011). It is hypothesized that it is in this region where the error signal between the model reference and this feedback is generated. Neurons from this region then project to the interomedial lateral nucleus of the spinal cord to produce vessel constriction, modifying peripheral resistance, which controls BP (DeStefino et al., 2011; Yates et al., 2014). Neurons in this region as well as axons from the glutamatergic neurons in RVLM also project to the Dorsal Motor Nucleus of the Vagus and the Nucleus Ambiguus to slow the heart (DePuy et al., 2013; Stornetta et al., 2013). It should be noted that the heart receives inhibitory input from the vagus nucleus separate from RVLM, but there is also an axon that projects from glutaminergic neurons in RVLM to the Dorsal Motor Nucleus of the Vagus (DePuy et al., 2013). This would cause a reduction in HR at the same time that projections to the InteroMedial Lateral portion of the cervical spinal cord are activated through RVLM to produce an increase in BP and HR through the sympathetic system. RVLM is indeed a major input to the sympathetic system in the spinal cord. It receives glutamatergic input from the vestibular (otolith) system (Holstein et al., 2012, 2014; Yates et al., 2014), which is insensitive to levels of alertness since VVRs can be induced in anesthetized rats. It also receives input from the parabrachial nucleus that, in turn, receives input from the hypothalamus and other cerebral hemispheric inputs. The model realization shown below considers this feedback loop as an important component of not only controlling BP, but also HR, but the specific control of HR has not been modeled as this is beyond the scope of this study.

### Model realization

**Figure 3:**
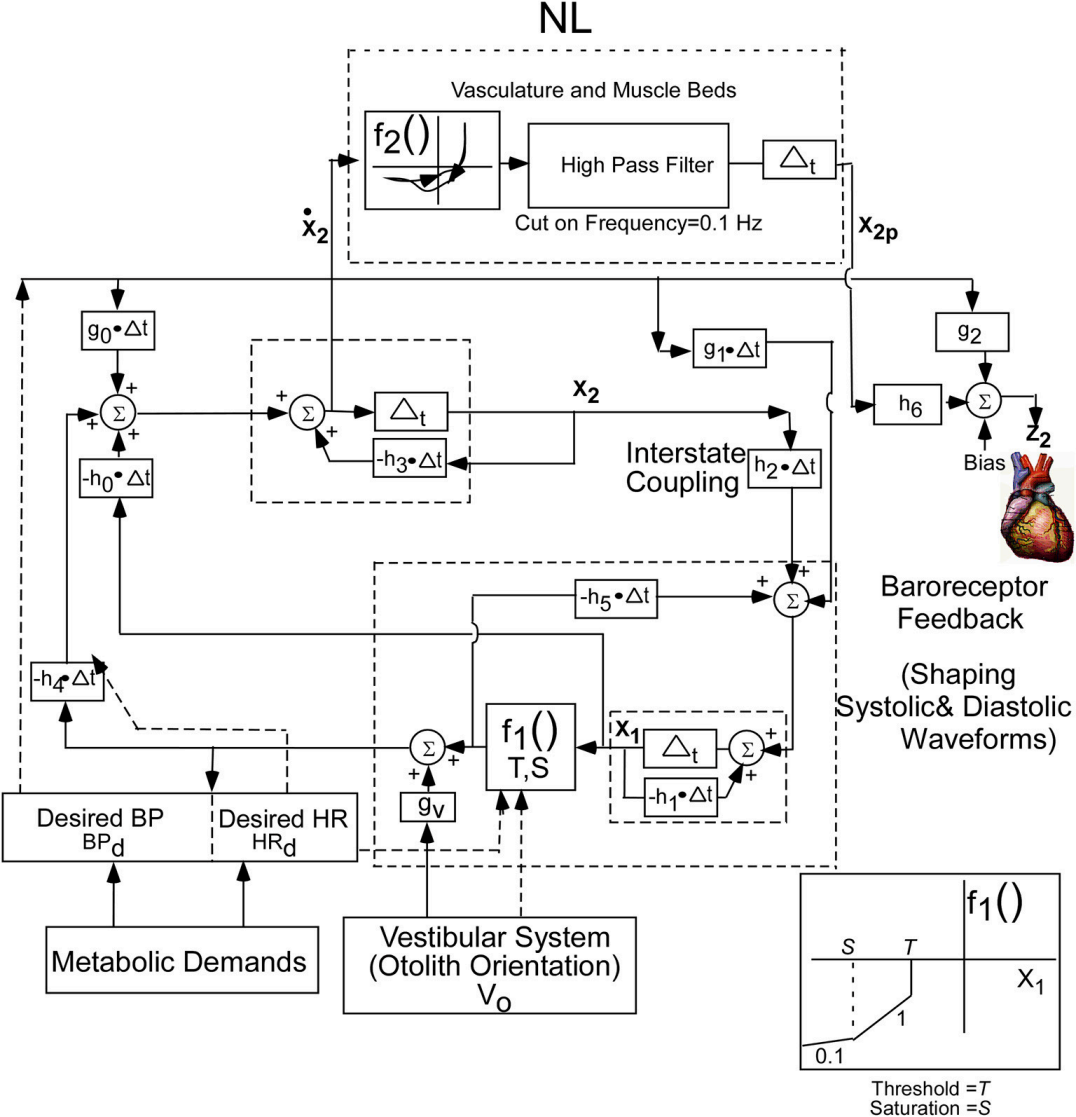
**Realization of the internal reference model as a second order relaxation oscillator**. Equations that implement the model were used to simulate BP and HR. The model parameters were chosen as:h0 = −40.0, h1 = 19.5, h2 = 35.6, h3 = 46.15, h4 = 140, h5 = −61.4, h6 = −0.05, bias = 30, g0 = −88.05, g1 = −18.5, g2 = 0.9, g3 = 0.1, S = −700, T = −40. The nonlinear function, NL, operated on the derivative of **x_2_** to generate the output reference signal for controlling the vasculature that controls BP. See text for model equations and detailed description.

The conceptual model was implemented by a second order system with two integrators whose outputs are the two states of the oscillator. This is a minimal structure for inducing relaxation oscillations. How these integrators are realized by central circuits is not known, but may be realized through functional commissural connections between RVLM's and NTS's that have been noted in Blessing (1997) and Granata (2003). In order to fit the data, a nonlinear function (NL) of the derivative of the first integrator state (**x_2_**) was utilized to drive BP (Figure 3). The nonlinear function was comprised of nonlinear enhancing function, f_2_, which enhances the slope of the derivative and contains a hysteresis effect that has a greater slope at the rising edge of the derivative of BP than the declining edge. The shape of the nonlinearity was determined by comparing the derivative of the BP data with those of the model variable behavior to optimize the fits by a two segment fourth degree polynomial fit to the data. The two segment polynomial enhancement of the derivative of **x_2_** captured the enhanced slopes and the hysteresis effect. The output of this nonlinearity was processed by a high pass filter with a cut-in frequency of 0.1 Hz (Figure 3) and then processed by an integrator, whose output has been labeled **x_2p_**. This output combines with Desired BP through parameters **h_6_** and **g_2_**, which is offset by a bias (Bias) to generate the signal **z_2_**, which is the reference signal for driving the vascular beds that control BP. The purpose of the high pass filter was to prevent drifts at the output of the integrator due to any DC component generated by the nonlinearity, f_2_. A Bias component regulated and constrained the level of the reference output for generating BP, z_2_ (Figure 3). The nonlinear function, NL, described above was characteristic of all of the data that we analyzed. The oscillating part of the relaxation oscillator model only had a few parameters so that the oscillatory behavior of the system could be easily studied (Figure 3). Thus, the oscillator maintained the systolic to diastolic transitions and the nonlinearity in the output shaped and constrained the BP and its derivative. For simplicity, only the effects of Desired BP were modeled and Desired HR was not considered.

The equations governing the relaxation oscillator and the output function are as follows:

(1) $$\left[\begin{array}{c} x_{1}(n+1)\\ x_{2}(n+1) \end{array}\right]\;=\;\left[\begin{array}{cc} 1+(-h_{1}-h_{5}\cdot f_{1}(x_{1}))\Delta t & -h_{2}\Delta t\\ (-h_{0}-h_{4}\cdot f_{1}(x_{1}))\Delta t & 1-h_{3}\Delta t \end{array}\right]\;\left[\begin{array}{c} x_{1}(n)\\ x_{2}(n) \end{array}\right]+\left[\begin{array}{c} g_{1}\Delta t\\ g_{0}\Delta t \end{array}\right]\;BP_{d}+\left[\begin{array}{c} 0\\ g_{v}\Delta t \end{array}\right]\;V_{0}(n)$$

(2) $$\begin{array}{ccc} z_{2}(n) & = & (-h_{6})x_{2p}+(g_{2})BP_{d}+\text{Bias} \end{array}$$

(3) $$\begin{array}{ccc} {\dot{x}}_{2p} & = & f_{2}({\dot{x}}_{2}) \end{array}$$

(4) $$\begin{array}{l} f_{1}(x_{1})=0\;x_{1}>T\;T=f(HR_{d},x_{1})\\ f_{1}(x_{1})=1\;x_{1}\leq T\\ f_{1}(x_{1})=0.1\;x_{1}\leq S \end{array}$$

Equation (1) represents the state equations of the oscillator. These are determined by two states, a nonlinear function, *f*_1_, governed by a threshold element, *T*, and a Saturation, S (Equation 4), feedback parameters, *h*_0_, *h*_1_, *h*_4_, and *h*_5_, feed forward parameters *g*_0_, *g*_1_, and *g*_*v*_, and inputs *BP*_*d*_ and *V*_0_. The input *V*_0_ represents a central otolith system signal, where sGVS and tilts of the head and body are converted to a command to the central baroreflex feedback that controls the relaxation oscillator. This input could modulate the amplitude of the systoles, and it has a similar effect as modulating Desired BP. The nonlinear function *f*_2_, in Equation (3), represent the polynomial enhancement of the derivative of **x_2_** and hysteresis. The parameters, *h*_6_, and *g*_2_, in Equation (2), represent the coupling of **x_2p_** derived from the oscillator and Desired BP to the output. The linear combination together with a Bias component determines the dynamic behavior of the systoles and diastoles.

The model has been implemented as an observable form realization in Matlab where the states of the model, **x_1_** and **x_2_**, are coupled to each other by parameter, **h_2_** (Zadeh and Desoer, 1963; Desoer, 1970). The basic function of the relaxation oscillator can be viewed as follows:

When the state **x_1_** is above a threshold, the system is over-damped and the state, **x_1_**, is driven to a negative steady level determined by its parameters described above. This establishes the diastoles. When threshold, **T**, is reached, additional feedback is activated which couples to the dynamical states through parameters **h_5_** and **h_4_**. The system becomes under-damped and induces an oscillation, so that **x_1_** increases slightly but then drops rapidly until it is above threshold. The energy of the oscillation at the initial stage of the systole carries the signal above threshold until it reverses. BP then falls to the diastole level and is retriggered when it crosses threshold, producing the next systole. The process repeats as long as it is driven by a constant signal, i.e., Desired BP. For an appropriate threshold, oscillations are sustained whose frequency and amplitude are determined by the Desired BP. The vestibular input, **V_0_**, is hypothesized to be a signal generated by the central otolith system summing with Desired BP through a gain, **g_v_**, which also modifies the frequency and amplitude of the systoles and diastoles. The vestibular input presumably also activates areas that generate Desired BP to reduce this variable and trigger a VVR. This mechanism has not been modeled, and is beyond the scope of this paper. However, simulations show that a drop in Desired BP induces a VVR.

An important parameter for characterizing the systolic-diastolic transition and for determining cardiovascular hypertension is the difference between the systolic and diastolic level or Pulse-Pressure (Domanski et al., 1999; Franklin et al., 1999; O'Rourke and Frohlich, 1999; Vaccarino et al., 2001). Model simulations help define how Pulse Pressure is affected by the various inputs and feedback parameters. Comparisons of model predictions with data on Pulse Pressure over a range of BP levels directly test the feasibility and predictive power of the model.

### Simulations of experimental data

**Figure 4:**
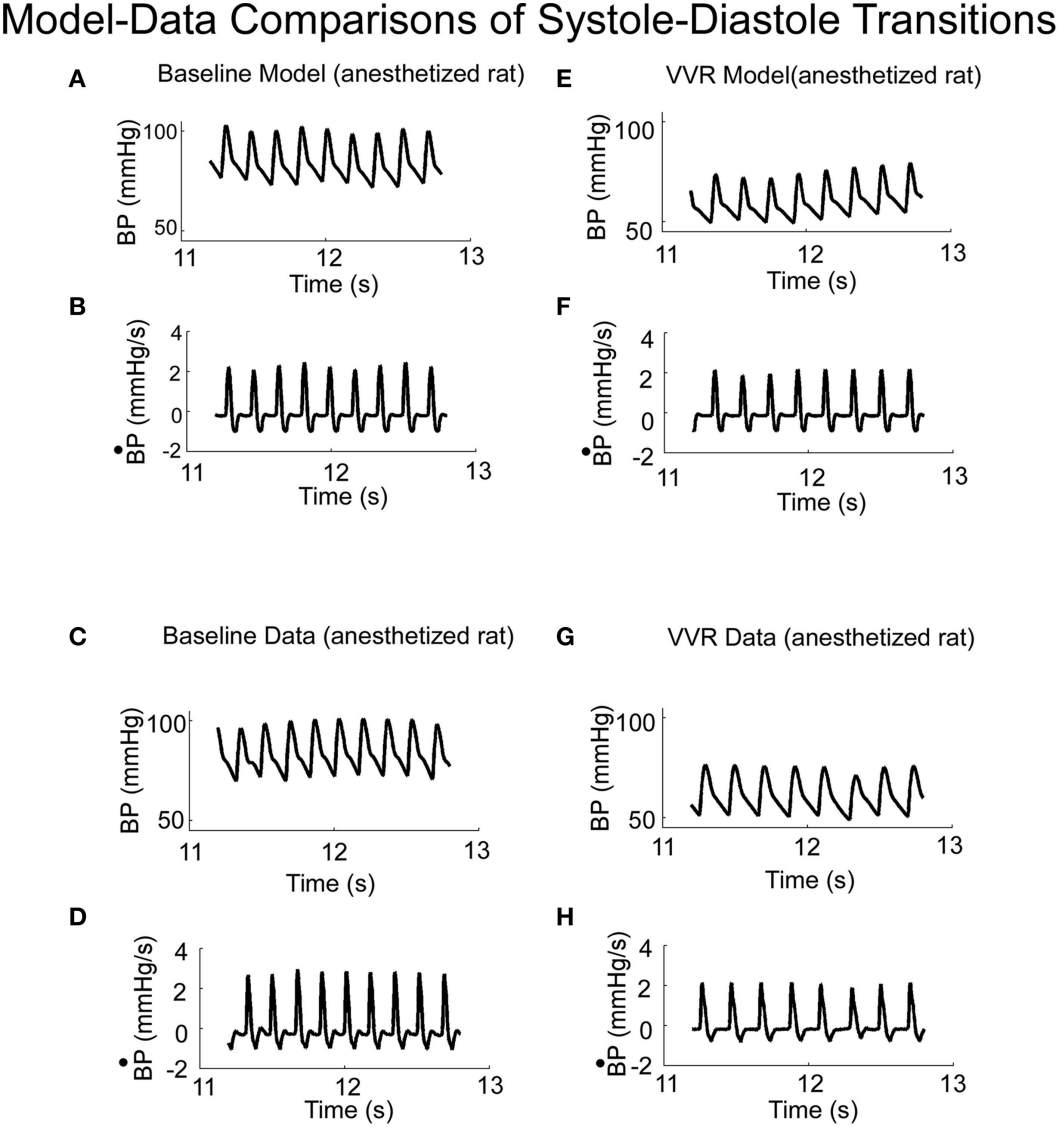
**(A)** Model predictions of systoles and diastoles during baseline BP. **(B)** Model predictions of the derivative of BP ($\overset{•}{\text{BP}}$). **(C,D)** Corresponding baseline experimental data of BP and its derivative, $\overset{•}{\text{BP}}$, measured in anesthetized rat. Model predictions of systoles and diastoles of BP **(E)**, and its derivative, $\overset{•}{\text{BP}}$ **(F)**, during a VVR. Note that with the NL function, the model predicted the average BP, systoles and diastoles acurately. **(G,H)** Corresponding baseline experimental data of BP and its derivative, $\overset{•}{\text{BP}}$, measured in an anesthetized rat.

When activated by a constant Desired BP, the output of the model oscillated at a fixed frequency (Figure 4A) and the systolic and diastolic phases compared favorably with those from an anesthetized rat when there was no external vestibular stimulus (Figure 4C). The model parameters could be adjusted to have an oscillator frequency close to that observed in the data (cf. Figures 4A,C), and the general character of the oscillations were close to that of the experimental BP data and its derivative. Thus, the key features of the systoles and diastoles in data were captured by this simple relaxation oscillator model. The model also predicted the functional relationship between size and frequency of systolic peaks under a wide range of conditions. When the model simulated a VVR by decreasing Desired BP, the peak to peak amplitude of each simulated systole was reduced (Figure 4E), its derivative was also reduced in peak to peak amplitude (Figure 4F) and the BP had the same properties as the experimental data (Figures 4G,H). Thus, the model had the flexibility to simulate experimental data in the normal state and during a VVR.

**Figure 5:**
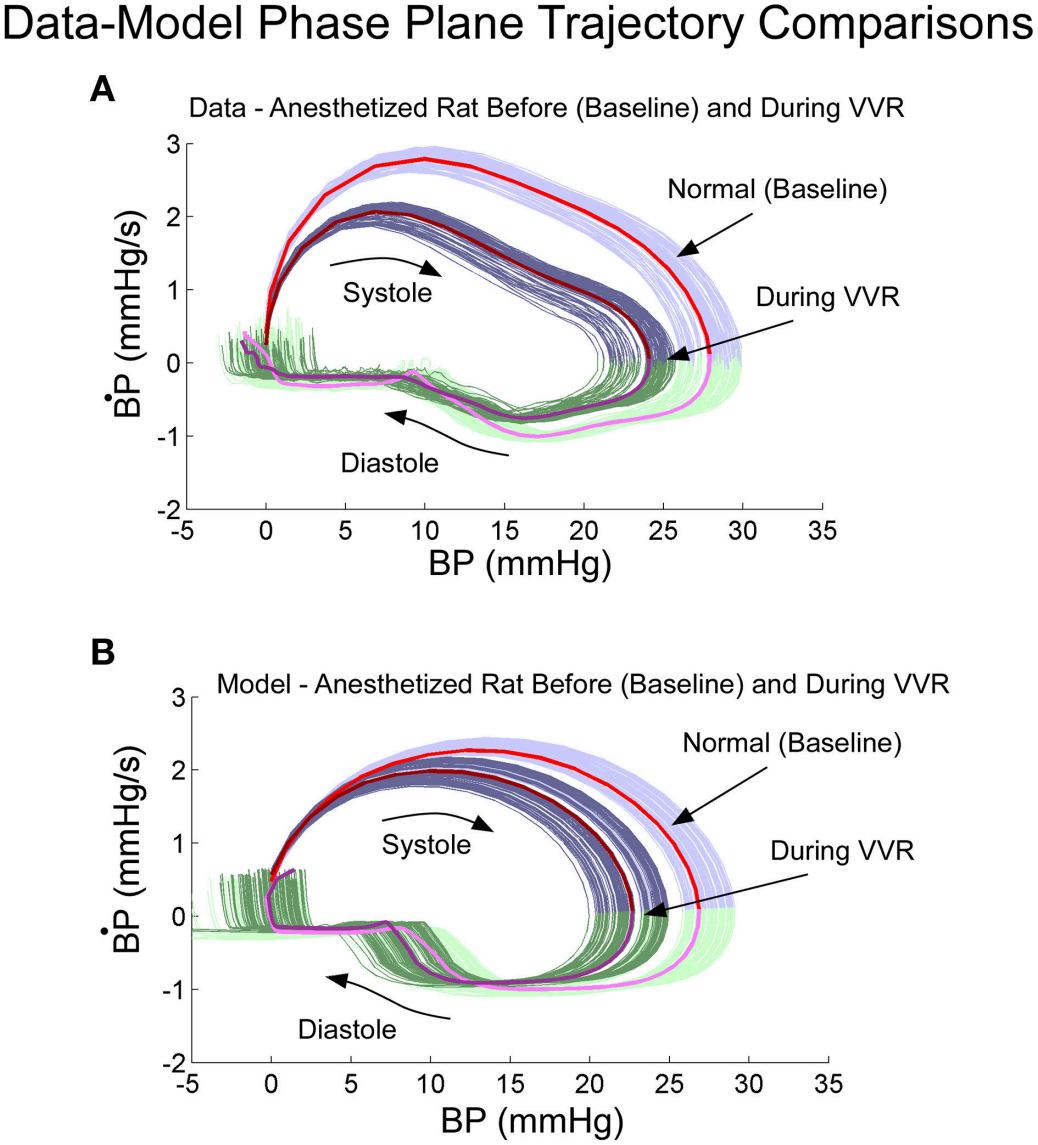
**Phase plane trajectory (B^·^P vs. BP) for experimental data (A) and model predictions (B)**. The shape of the predicted model trajectory accurately predicted the data both before and after a VVR. See text for explication.

Phase plane trajectories, which showed the rate of change of BP as a function of BP during normal states and during a VVR, were compared for model and systolic-diastolic transitions. Both trajectories were closed and reached a peak value for the derivative ~25% into the trajectory and then leveling off (cf. Figures 5A,B). The model had a rounder trajectory and did not have the inflection during the trajectory as did the data (Figures 5A,B). but considering that the relaxation oscillator part of model is only of second order, the nonlinearity was piecewise linear representing threshold and saturation, and the nonlinear function representing the output driven by a single variable was a two part polynomial followed by a simple high pass filter, the model predicted the phase plane trajectories in the anesthetized rat fairly accurately both during normal states and during a VVR (Figure 5).

**Figure 6:**
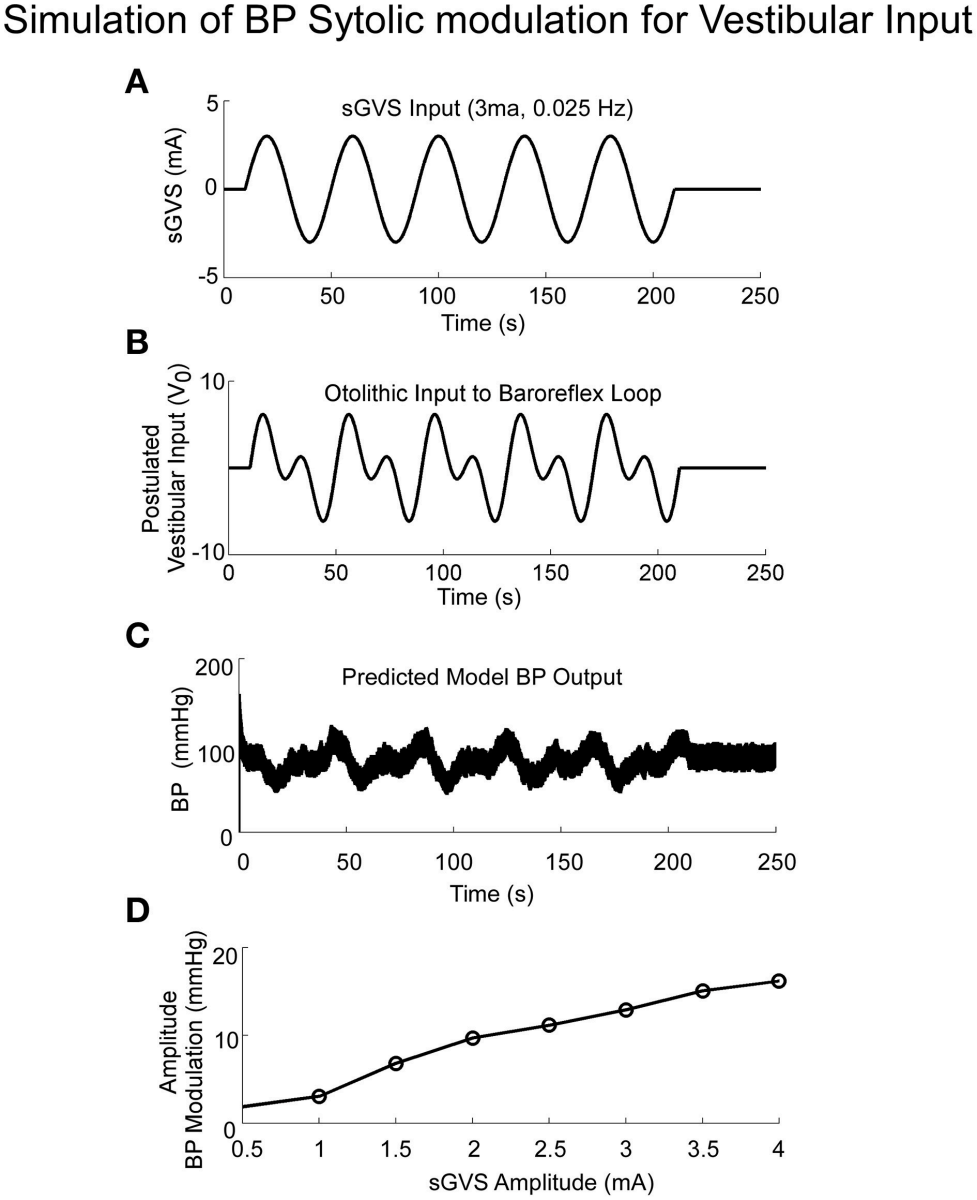
**Simulations of systolic BP modulation due to sGVS (A)**. The central otolithic signal that inputs to the baroreflex was assumed to have a second harmonic component **(B)**. **(C)**The model-predicted systolic modulation contains this strong second harmonic in accordance with the data. **(D)** The amplitude of systolic and diastolic modulations as a function of vestibular input.

The model simulations also predicted the increased amplitudes of low frequency oscillations in systolic BP, i.e., Vaso-Vagal Oscillations, which were induced by low frequency vestibular stimulation (Figure 6) and compared favorably with the experimental data (Figure 1C). In response to sGVS (Figure 6A), central otolithic activation was assumed to have a strong second harmonic component in addition to the primary harmonic (Grewal et al., 2009; Cohen et al., 2011, 2013; Yakushin et al., 2014; Figure 6B). This assumption enabled comparisons and evaluation of model performance with data. The model predictions, including the second harmonic component (Figure 6C), were similar to the Vaso-Vagal Oscillations observed in the data (Figure 1).The model also predicted increased modulation in systolic amplitudes due to increases in vestibular activation, essentially mapping the linear increase until a saturation level (Figure 6D). This essentially predicted the vestibular sympathetic response (VSR) for sinusoidal vestibular stimulation.

### Pulse pressure - independent test of the model

**Figure 7:**
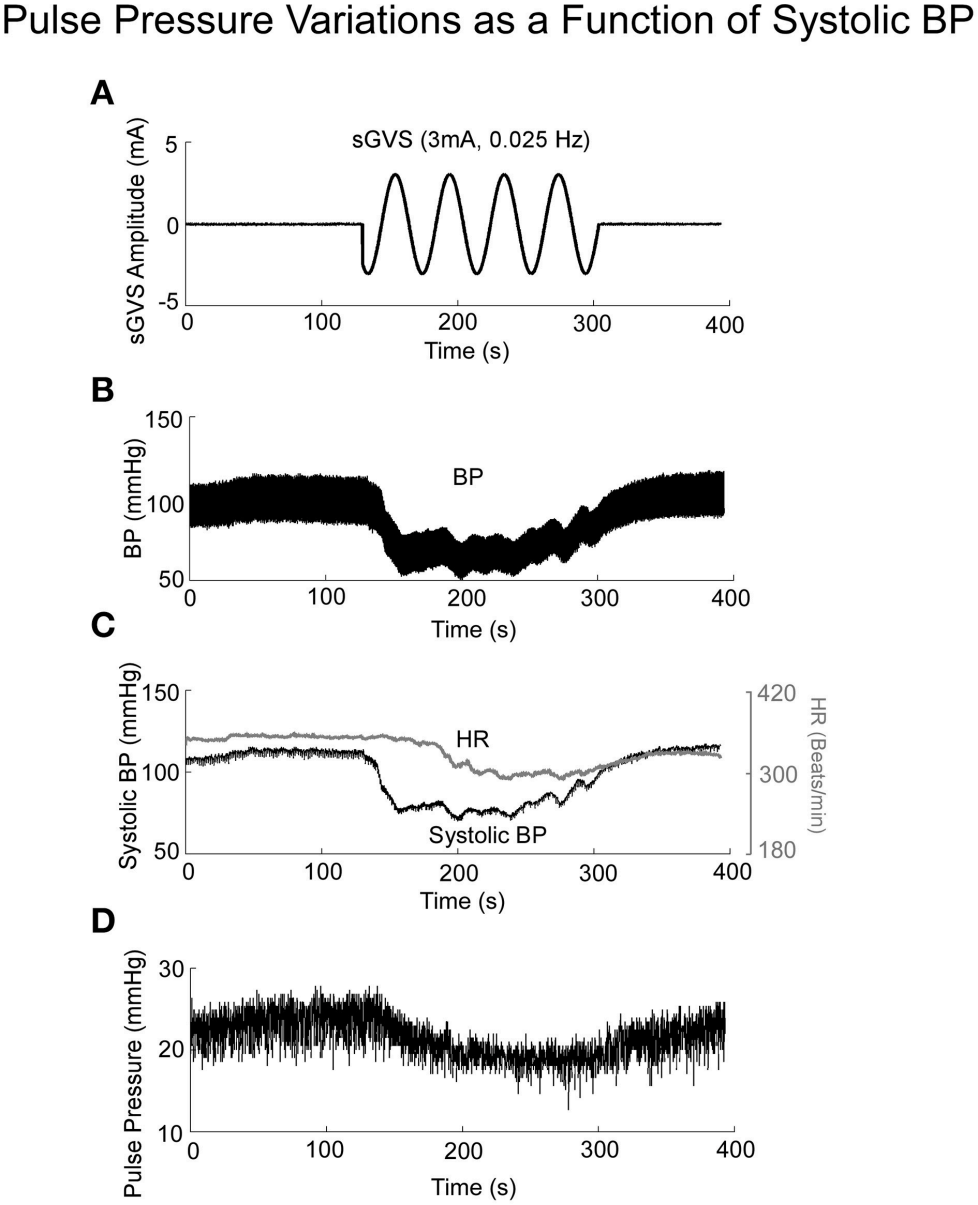
**BP, HR, and Pulse Pressure in response to 3 mA, 0.025 Hz sGVS**. **(A)** sGVS stimulus. **(B)** BP response showing a VVR and oscillations in response to sGVS. **(C)** Systolic BP as a function of time, before, during, and after sGVS. BP transiently fell, was sustained during stimulation and then rose back to steady state level. HR also fell, but not synchronously with BP. **(D)** Pulse pressure averaged over 25 s interval rose and fell in synchrony with BP.

**Figure 8:**
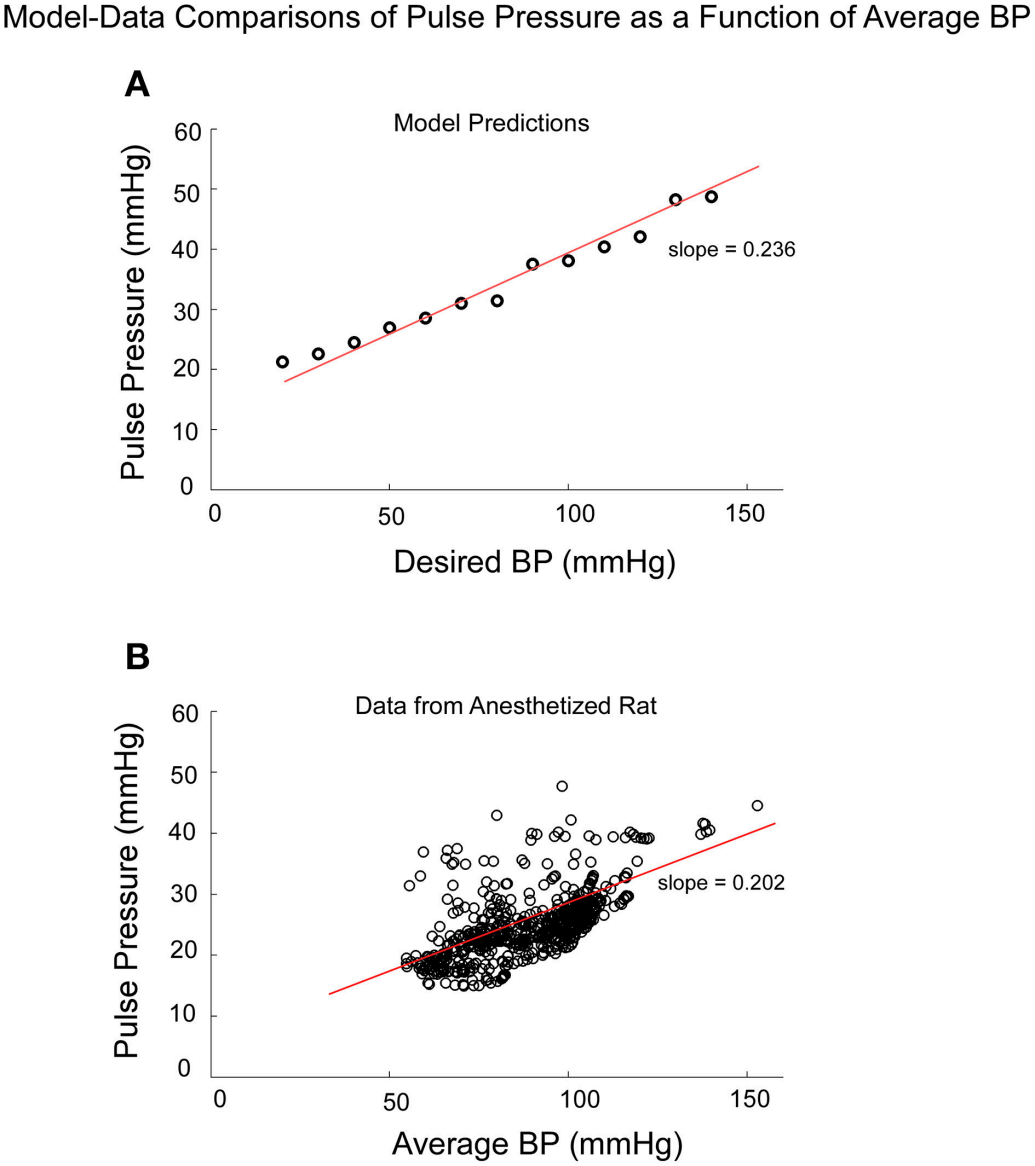
**Comparison of model predictions of Pulse Pressure vs. Desired BP (BP_*d*_) and data of Pulse Pressure vs. Average BP from an anesthetized rat**. Average BP was assumed to be an estimate of Desired BP **(A)** The model predictions of Pulse Pressure were approximately linearly related to Desired BP as pule pressure ranged from 20 to 50 mmHG. Pusle Pressure then saturated due to the saturation representing the baroreflex feedback. **(B)** The experimental data also had an approximate linear increase as a function of Average BP. The available data were obtained for Average BP ranging from 50 to 120 mmHG. Data points at 140 and 160 mmHG Average BP also followed this linear trend.

If Pulse Pressure were to be predicted by the simulations, it would be an independent test of the underlying concept. Pulse Pressure, which is the difference between systolic and diastolic levels, approximately represents the force that the heart generates each time it contracts (Franklin et al., 1999; Vaccarino et al., 2001). In response to sGVS during a typical experiment (Figure 7A), BP generally rose and then dropped dramatically during a VVR (Figure 7B) and then rose again slowly to steady state levels. For this particular experiment, systolic levels rose slightly prior to initiating the sGVS (Figure 7B), although HR remained level (Figure 7C). The rise in BP prior to sGVS was variable and did not always occur. At the initiation of sGVS, systolic BP dropped transiently while HR slowly declined at a later time (Figure 7C). Both Systolic BP and HR then returned to steady state levels. Pulse Pressure followed the trend of systolic BP, although the drops in pulse pressure were somewhat slower (Figure 7D). A key prediction of the model was that Pulse Pressure would be approximately proportional to systolic BP, which is approximately proportional to Desired BP. This prediction compared favorably with the experimental data. When simulations were run with different levels of Desired BP, the model predicted an approximately linear increase in pulse pressure with Desired BP (Figure 8A). The data also showed that pulse pressure rose approximately linearly with Average BP (Figure 8B), when average BP was computed over a 25 s window. The differences could be because in the model the Desired BP could be precisely controlled, whereas the data were lumped with those observed during VVR to expand the range over which Average BP could be plotted. There are also probably nonlinearities in the processing that were not considered. Despite small differences, this simple model predicted the experimental data on pulse pressure, and demonstrated the predictive capability of the model. This result also suggests that pulse pressure might be an important parameter when considering cardiovascular function.

**Figure 9:**
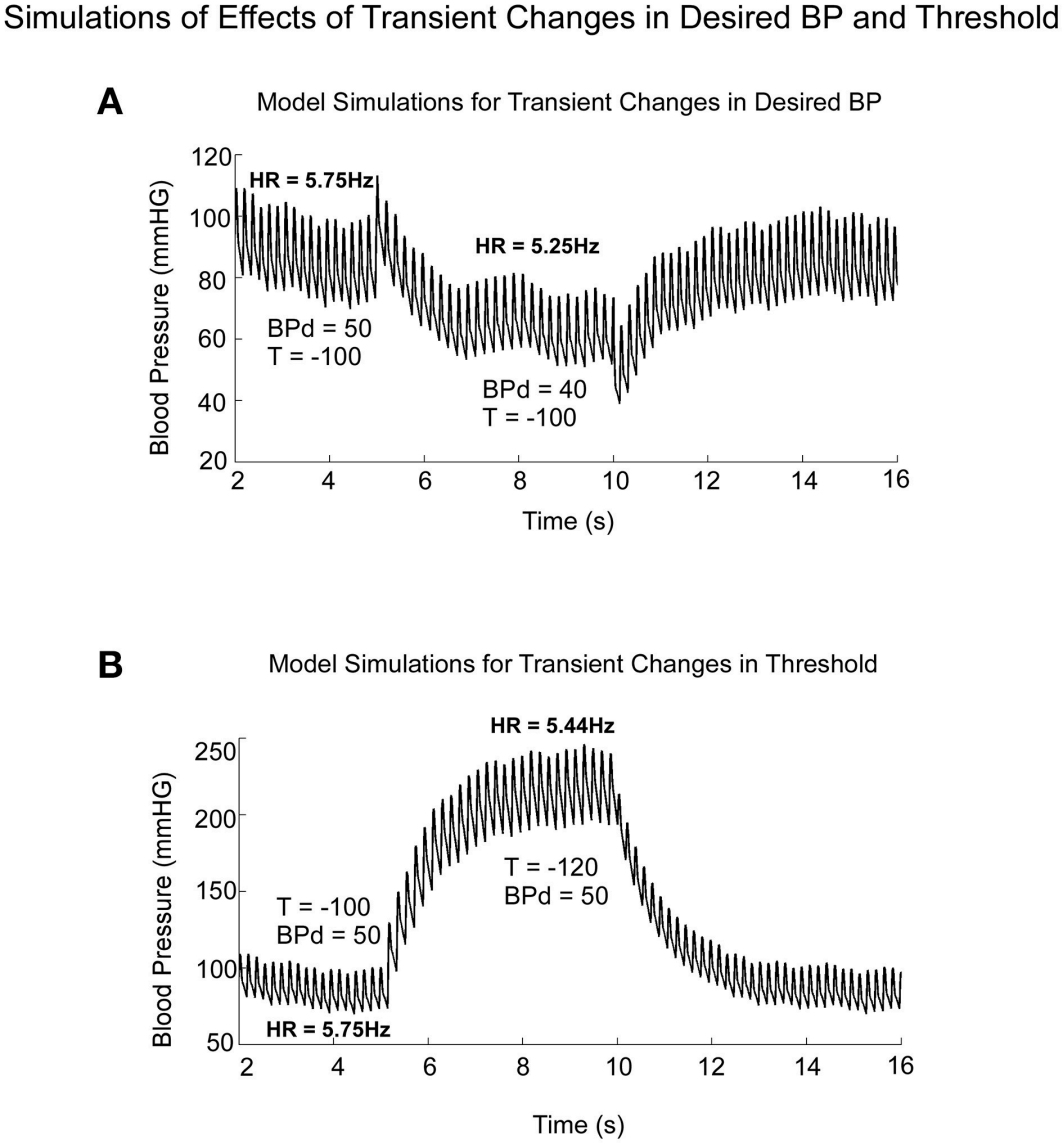
**Model predictions of the differential effects on the systolic to diastolic transitions as a function of time due to transient changes in Desired PB and Threshold (T)**. **(A)** Changes in Desired BP simulated the anticompensatory behavior of BP and HR during a VVR with drops in BP, Systolic BP, Pulse Pressure, and HR. **(B)** Changes in Threshold generate drops in HR, with increases in time between systoles (R-R interval). During the transient change in Threshold, there were large increases in Systolic BP and pulse pressure, not consistent with a VVR.

Model simulations clearly demonstrated the difference in BP and HR transitions in Desired BP and Threshold (**T**; Figure 9). A transient drop in Desired BP (50–40 mmHG) (Figure 9A) induced a drop in average BP, HR, and Pulse Pressure (Figure 9A), similar to changes that occur during a VVR (Figures 1, 7). The transient drop in BP while maintaining the threshold *T* = −100, produced smaller systolic levels and decreased HR from 5.75 to 5.25 Hz (Figure 9A). A transient drop in threshold while maintaining BP_d_ = 50, also reduced HR (Figure 9B), but this was compensated by increases in systolic levels of BP and Pulse Pressure, which tended to maintain the average BP (Figure 9B).

**Figure 10:**
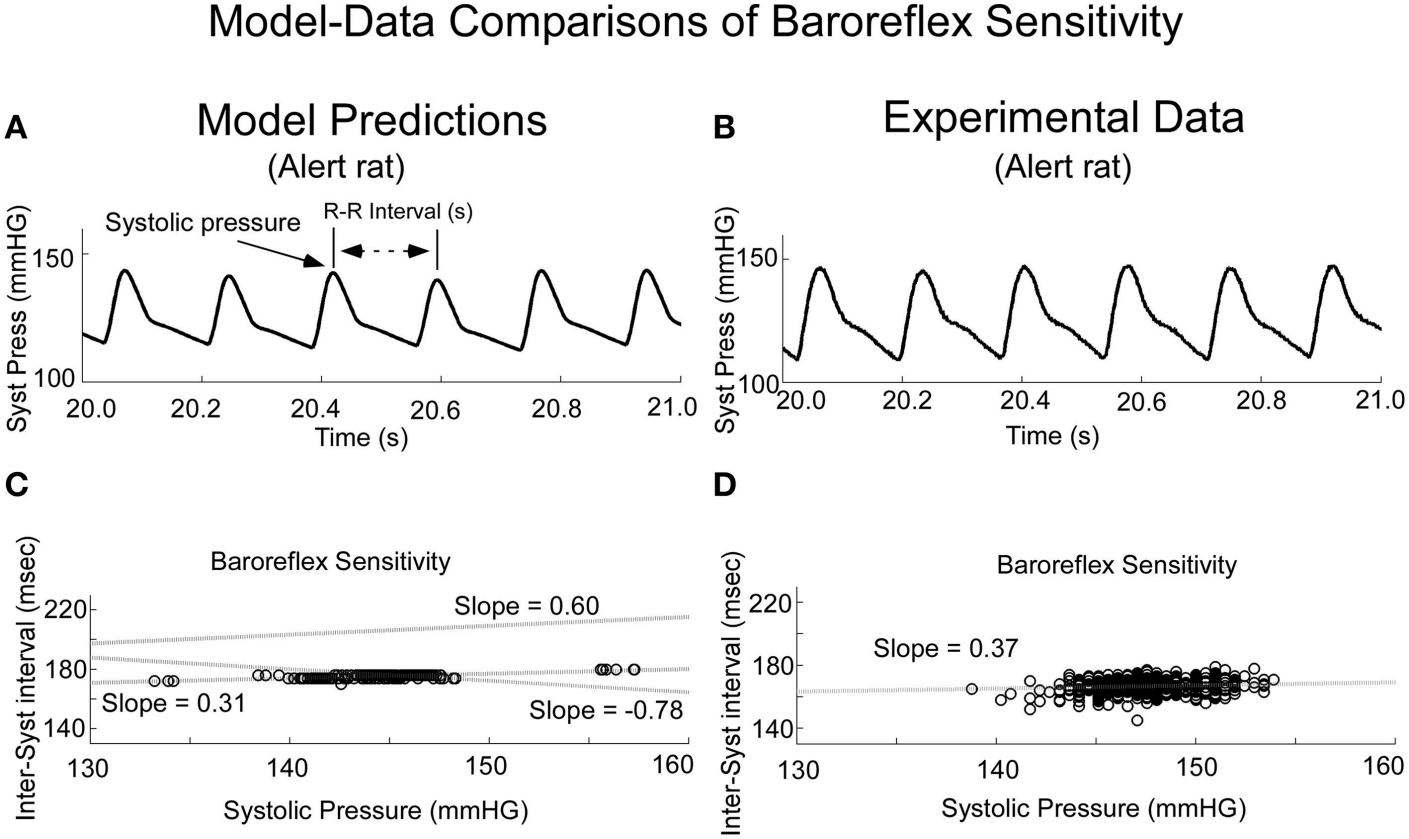
**Comparisons of model predicted Baroreflex Sensitivity with that derived from experimental data**. **(A)** The model predicted variations in frequency of systoles, the inverse of HR. **(B)** Experimental data in alert rats also had this variation in frequency of systoles. **(C)** The model simulated Baroreflex Sensitivity by varying the threshold, but by altering the level of the vestibular input, the slope could be changed (dotted lines with varying slopes). **(D)** The Baroreflex Sensitivity in the alert rat also had a positive slope, which was similar to that of the model prediction with no vestibular input. This slope was somewhat greater than the slope for the anesthetized rat, which was close to zero.

When variations in threshold were considered, the model predicted the behavior, which has commonly been referred to as baroreceptor sensitivity (Davos et al., 2002), which is defined as R-R interval as a function of previous Systolic Pressure (Figure 10). When the threshold, **T**, was varied randomly, the model output achieved varying systolic levels (Figure 10A). This was the approximate behavior in the systolic levels in the alert rat (Figure 10B). When subsequent R-R interval was plotted as a function of Systolic Pressure (Baroreceptor Sensitivity), the model predicted a positive correlation for the Baroreceptor sensitivity for vestibular input V_0_ = 0 **(**Figure 10C, **Shaded dotted line, slope** = **0.31)**. This compared favorably with the positive correlation between the inter-systolic intervals and systolic pressure in the alert rat (Figure 10D). With appropriate vestibular input, the model predicted a rather marked change in slope. For a constant input V_0_ = 10, the slope was increased to 0.6, which ranged to the baroreceptor in humans (Davos et al., 2002). When a sinusoid of frequency of 0.025 Hz was applied as vestibular input, the slope was inverted to −0.78 (Figure 10C, **Slope** −**0.78)**, which may be a contributing factor in generating a VVR. With different vestibular inputs, the model could predict the slope in the data of the anesthetized rat, which was close to zero (not shown). Thus, when we adjusted the stochastic variation in threshold and vestibular input, a range of Baroreceptor Sensitivities could be obtained that were similar to those found in alert and anesthetized rats as well as humans (Davos et al., 2002). An exact match to the data for alert and anesthetized rats would require the development of nonlinear identification schemes and was beyond the scope of this study. Also, the range of systolic pressure was maintained within the limits of the data.

Thus, the model simulations indicated that Desired BP is a critical signal in initiating a VVR as both systolic BP and HR dropped as a result of its transient reduction. Modulations in threshold could decrease **HR**, but did not appear to play a major role in initiating the VVR, as Baroreceptor sensitivity was maintained.

## Discussion

The major contribution of this paper is the development of a mathematical model using a relaxation oscillator to simulate systolic and diastolic waveforms of the anesthetized rat, both at rest and during the VVRs that underlie VasoVagal Syncope. Fundamental to the oscillating system was the heart pump that generates systoles and diastoles. It was postulated that this oscillating heart pump was represented by a neural implementation of a relaxation oscillator, which was controlled by feedback and feed forward neural circuitry that ensured that the oscillating pump (heart) worked in conjunction with the demands of other reflexes such as the baroreflex and the VSR. The internal model mimicked the rhythmic behavior of the systoles and diastoles associated with the heart during normal and pathological function. Specifically, the model simulated the VSR (Yates and Miller, 1994; Yates, 1996; Yates et al., 2014), as well as how VVRs could be initiated from vestibular stimulation by reducing Desired BP, and how pulse pressure might be controlled centrally. An emergent property of the model was that it simulated the low frequency Vaso-Vagal oscillations associated with a VVR. The model also simulated the Baroreflex Sensitivity, which is a fundamental component of the baroreflex feedback, by varying the threshold and calculating the slope of the scatterplot of the inter-systolic interval vs. systolic level. The use of a threshold and saturation and a second order system was essential for the implementation of the model as a relaxation oscillator. Thresholds and saturation have been a cornerstone of models of the baroreflex, which is a fundamental feedback circuit controlling BP (Chen and Kuo-Chu, 1991; Ottesen, 2000; Chen and Shi, 2011; Ottesen and Olufsen, 2011; Mahdi et al., 2013). Adding stochastic variations to the threshold in the feedback loop not only simulated the low frequency variations in systolic levels referred to as Mayer waves (Cheng et al., 2004; Julien, 2006), but also demonstrated that the model naturally implemented the Baroreceptor Sensitivity, which has been an important feature when describing cardiovascular regulation (Davos et al., 2002; La Rovere et al., 2008). Other feedback mechanisms besides the baroreflex may also play a role. Thus, by simulating effects of Desired BP, threshold, and saturation, the model explained that a drop in Desired BP was most likely responsible for inducing VVRs. On the other hand, effects of threshold variation brought into evidence the function of Baroreceptor Sensitivity. By coupling the otolith system to the baroreflex feedback, the VSR could be activated, simulating the oscillations in systolic levels of BP and HR when anesthetized rats were exposed to sGVS and tilts (Cohen et al., 2011, 2013; Yakushin et al., 2014). A striking finding was that the slopes of the Baroreceptor Sensitivity could be changed by vestibular otolithic input (Figure 10C) and that a low frequency sinusoidal otolithic input could actually invert the slope of the Baroreceptor Sensitivity function. This kind of behavior could contribute strongly to the generation of a VVR that was induced by the sGVS and tilt stimulation.

Similar waveforms to those generated by a relaxation oscillator are not unique to the cardiovascular system, as they are found in other motor systems as well. These include the system that generates the slow and quick phases of vestibular and optokinetic nystagmus (Raphan, 1976; Cohen et al., 1977, 1987; Raphan et al., 1979; Waespe et al., 1983) and the swing and stance phases of locomotion in both monkeys (Xiang et al., 2007) and humans (Osaki et al., 2007, 2008; Cho et al., 2010). This is the first time such a model has been used to study the systolic to diastolic transitions and to simulate a VVR, to our knowledge, however.

An important consideration was whether feedback from cardiovascular mechanisms has the threshold and integrative properties to modulate relaxation oscillations in BP and HR. While the heart can generate systoles and diastoles without feedback, the lack of such feedback would not allow the heart to respond appropriately to the demands that require rapid modulation of BP and HR. The baroreflex has the appropriate pressure sensor in the aorta and carotid sinus to provide the feedback signal that central mechanisms need to control BP and HR. The baroreflex also has the nonlinear threshold and saturation functions (Chen and Kuo-Chu, 1991; Ottesen, 2000), which have been modeled by a sigmoidal function (Ottesen, 2000; Mahdi et al., 2013). This nonlinear function is the switch that maintains the relaxation oscillations in the model reference. When the threshold is exceeded by the feedback signal, the system enters a systolic mode and when the feedback signal is below threshold, the system is in the diastolic mode. The continuous modulation of MSNA with each systole and diastole (Eckberg, 1977; Wallen and Sundlöf, 1982; Mosqueda-Garcia et al., 1997; Voustianiouk et al., 2006) is evidence that the heart and arterial and vascular beds are being controlled. The saturation level for the nonlinear feedback element ensures that pulse pressure remains within bounds so as not to damage the vessels that carry the blood flow. Other feedback mechanisms may exist to produce the temporal variations that generate systolic and diastolic behavior, but as yet they are unknown.

Previous models used linear input-output oscillators to simulate variations in BP (Bertram et al., 1998; Julien, 2006) and did not consider the modulation of the existing oscillating system, i.e., the heart pump). Linear oscillators rely on positive feedback, so that the loop gain is close to unity. This creates instability so that noise in the system would cause the system to oscillate close to a single frequency (Barkhausen, 1935). Systolic frequencies continuously shift, however, making it impossible for a linear oscillator to represent either BP or HR accurately. BP also does not vary sinusoidally, but the changes are triangular (Figure 1A), so the rhythmic systolic to diastolic transitions cannot be produced by a linear oscillator. The relaxation oscillator used as the basis for the proposed model generates triangular waves whereby a diastole is a relaxation from a systole, rather than just a rapid drop in BP. The control of the oscillator is accomplished by modifying parameters that are capable of modulating the amplitude and frequency of the nonlinear oscillator. If the feedback from baroreceptors generates an error between the actual BP, HR, and fluid flow in the arteries relative to the model reference variables, this error is corrected by adjusting the size of the arterial bed, HR and ultimately the fluid flow in the arteries and veins.

Smooth functions, such as a logistic function, have been used to model the baroreflex transformation (Chen and Kuo-Chu, 1991; Ottesen, 2000; Ottesen and Olufsen, 2011), which presumably is responsible for generating the error signal between actual BP and HR from those generated by the model reference. This nonlinearity was too smooth to accurately predict the systolic and diastolic transitions and phase plane trajectories, however. The piecewise linear approximation, used to model the threshold and saturation, had more abrupt transitions and gave better results in approximating the phase plane plots and shapes of the systoles (Figure 5). This suggests that better approximation functions need to be considered when modeling the baroreflex feedback (Ottesen, 2000) that are more in line with the piecewise linear approximation functions presented here.

An important consideration derived from the simulations was the relative effect of Threshold and Desired BP on the generated BP and HR, since both are needed to sustain the relaxation oscillations. When the magnitude of the threshold was raised to determine its effect on BP without a saturation element, there was a decrease in the frequency of the systoles. However, the amplitude of the systoles and pulse pressure became very large. Thus, threshold changes were not critical for initiating a VVR, since blood volume could be sustained by generating larger systoles at a decreased frequency with larger pulse pressures (Franklin et al., 1999). The saturation incorporated in the model that limited pulse pressures in accordance with the experimental data (Figure 9) could be a safety mechanism so as not to damage arterial walls (O'Rourke and Frohlich, 1999). Of interest is that by varying the threshold stochastically, low frequency modulations in the amplitude of the systoles were induced, similar to those described as Mayer waves (Vielle, 2005; Julien, 2006) and the low frequency VasoVagal oscillations associated with VVR generations (Yakushin et al., 2014). The model predicted that under these circumstances, larger systolic BPs generated larger subsequent intersystolic intervals. i.e., a lower HR, which has been taken as the compensatory Baroreflex Sensitivity (Davos et al., 2002; La Rovere et al., 2008).

A critical question is how VVRs are generated with combined drops in both BP and HR. The model predicted that a decrease in Desired BP lowered the amplitude of the systoles, thereby reducing their frequency and the pulse pressure (Figure 9). This indicates that it was the drop in Desired BP, not changes in the Threshold that had initiated the VVR. This shows that if the central signal that generates Desired BP is maintained, compensatory mechanisms associated with Baroreflex Sensitivity could maintain adequate blood flow. If there is a drop in Desired BP, however, then a combined fall in BP and HR sends the oscillator into a reset state, i.e., a VVR. Where the signal related to Desired BP is generated in the central nervous system and how it functions is as yet not known.

An important question is how the vestibular system couples to the oscillating system to initiate a VVR. In the model, central otolith signals input to the feedback path through parameter g_v_, after the threshold-saturation element and before summating with the signal representing Desired BP. Thus, the vestibular input can alter the amplitude of the systoles, thereby modulating BP and HR to implement the VSR. The model also suggests that continuous oscillation induced by this input can generate a precipitous drop in **Desired BP**, simulating the behavior during VVRs, presumably through cerebellar mechanisms (Julu et al., 2003; Grubb, 2005).

Connections between the vestibular and autonomic systems and the baroreflex in RVLM are consistent with the structure and function of the model. RVLM receives direct glutamatergic input from otolith regions of the vestibular nuclei (Yates and Miller, 1994; Holstein et al., 2012, 2014; Yates et al., 2014), which projects to the adrenergic system in the spinal cord. RVLM also gets inhibitory feedback from NTS via the baroreflex input directly and disynaptically through CVLM (Sved et al., 2000, 2001; Schreihofer and Guyenet, 2002; Sugiyama et al., 2011). The study of Gotoh et al. (2004) in alert centrifuged rats before and after baroreflex denervation demonstrated that changes in BP were much larger after deneravation of the baroreflex. This suggests that the baroreflex has an inhibitory effect on the VSR. The RVLM, which is located on both sides of the medulla, also has inputs from the parabrachial nuclei (Balaban, 1996; Len and Chan, 1999; Balaban et al., 2002), that get input from the hypothalamus, entorhinal cortex and other regions of the cerebral hemispheres (Felder and Mifflin, 1988; Balaban et al., 2002; Bowman et al., 2013). Thus, the baroreflex and vestibular input merge in RVLM, which is consistent with the hypotheses proposed in the model. There is also a projection to the Dorsal Motor Nucleus and to Nucleus Ambiguus, which would provide inhibitory input to the heart.

Less obvious is the origin and operation of the reference signal, Desired BP. A likely source is the uvula, which has projections to the otolith regions of the vestibular nuclei, the parabrachial nuclei and disynaptically to NTS to interact with the baroreflex (Bradley et al., 1990; Paton et al., 1991; Silva-Carvalho et al., 1991; Balaban et al., 2002; Nisimaru, 2004). Purkinje cells in the uvula have modulations of firing rates dependent on head position relative to gravity (Tsubota et al., 2012). If the uvula generated Desired BP, it could also initiate a drop in Desired BP, which is likely to be the most important driver in generating a VVR. The uvula may also have a wider control of cardiovascular function through parameter adaptive regulation by receiving not only information from the vestibular nuclei, but also from the states of the integrators in the neural reference model that determine the patterns of BP and HR.

Two neural integrators have been postulated by the model to implement the relaxation oscillations. As yet, neither has been identified. Brainstem integrators have been simulated by neural net models for vestibular and oculomotor systems (Anastasio and Robinson, 1989; Anastasio, 1991). They have been realized by commissural connections between the vestibular nuclei on either side of the medulla, as in the vestibulo-ocular reflex (Galiana et al., 1984). Such integration disappears after commissural pathways between the medial vestibular nuclei on both sides of the brainstem are separated (Katz et al., 1991; Wearne et al., 1997). Furthermore, the integration is dependent on the integrity of the crossing axons of GABAergic neurons in the medial vestibular nuclei (Holstein et al., 1999a,b). There is a commissural pathway between the RVLM's on either side of the medulla (Granata, 2003). This pathway could potentially be part of one integrator in the feedback loop postulated by the relaxation oscillator model. Whether such integration is also GABAergic, as in the vestibulo-ocular reflex, is not known, but there are GABAergic neurons in the RVLM that could perform this function (Schreihofer and Guyenet, 2002; Guyenet, 2006). There is also a commissural system between the Nuclei Tractus Solitariei on both sides of the brainstem (Blessing, 1997). A portion of the commissural system, the Caudal Pressor Area (CPA), has both GABAergic and glutaminergic inputs, and can produce hypertension for prolonged periods (Sved et al., 1985; Otake et al., 1992; Takakura et al., 2007; Favero et al., 2011). It is unlikely that it accesses RVLM, but the specific pathways to the spinal cord to implement the hypertension are still to be defined.

When developing the model, there were considerations as to which states of the relaxation oscillator would best fit the data. Neither state **x**_2_ or **x**_1_ nor their derivatives predicted the average trajectories before and during a VVR (Figures 4, 5). Therefore, a nonlinear function of the derivative of state **x_2_** was used to drive the output and generate the control of BP. It should be noted that this derivative is obtained by the appropriate feedback loops in the model. The nonlinear function enhanced the derivative of **x**_2_ by a fourth order polynomial with hysteresis to sharpen the rise of the derivative of BP and slow the drop in BP. This signal was then high pass filtered to avoid drift and could be used to adjust the vascular beds that control BP. How and where such nonlinear functions are realized centrally is not clear, but the model structure has identified the types of function such as polynomial enhancement, hysteresis, and high pass filtering of relaxation oscillator variables that should be implemented to generate appropriate trajectories. The model has also identified neural integrators in the generation of systoles and diastoles, which could be related to the RVLM commissure, which has hitherto not been considered. The model has also enabled study of the events that trigger the transient decline in BP and HR during VVRs. It has focused on Desired BP as an important variable that controls BP and HR and has elucidated the consideration of pulse pressure in cardiovascular evaluation. These insights suggest how the Vestibular System might couple to the oscillator to control BP and HR to generate vasovagal oscillations. It could enhance considerations about how to predict modulation in BP from other modalities besides the vestibular system. Finally, it has identified key parameters that could be involved in the generation of the VVR that underlie Vaso-Vagal Syncope.

## Author contributions

TR contributed the overall conceptual framework for the study. He was responsible for developing the model and the organization and writing of the manuscript. BC contributed to the writing of the manuscript. He also contributed to the ideas concerning the relationship of the model to physiological function. YX contributed to the development of the implementation of the model and the comparison of model output with the data. He also contributed to the writing of the manuscript. SY contributed to the design of experiments and data acquisition that was important for comparing the data to the model. He also contributed to the writing of the manuscript.

### Conflict of interest statement

The authors declare that the research was conducted in the absence of any commercial or financial relationships that could be construed as a potential conflict of interest.

## Abbreviations

BP: blood pressure
Desired BP: Desired Blood Pressure
HR: heart rate
MSNA: Muscle Sympathetic Nerve Activity
sGVS: sinusoidal Galvanic Vestibular Stimulation
VVR: VasoVagal Response.

## Definitions

Desired BP: A presumed centrally determined reference signal to maintain BP
Pulse Pressure: Difference between systolic BP and Diastolic BP
Relaxation Oscillator: A nonlinear oscillator producing a non-sinusoidal output
Plant: heart and the peripheral vasculature.
